# Systematic altering of semiflexible DNA-based polymer networks *via* tunable crosslinking†

**DOI:** 10.1039/d2nr05615a

**Published:** 2023-04-05

**Authors:** Martin Glaser, Paul Mollenkopf, Dusan Prascevic, Catarina Ferraz, Josef A. Käs, Jörg Schnauß, David M Smith

**Affiliations:** a DNA Nanodevices Group, Fraunhofer Institute for Cell Therapy and Immunology Perlickstr. 1 04103 Leipzig Germany david.smith@izi.fraunhofer.de; b Soft Matter Physics Division, Peter Debye Institute, Faculty of Physics and Earth Sciences, Leipzig University Linnéstr. 5 04103 Leipzig Germany

## Abstract

In order to understand and predict the mechanical behaviours of complex, soft biomaterials such as cells or stimuli-responsive hydrogels, it is important to connect how the nanoscale properties of their constituent components impact those of the bulk material. Crosslinked networks of semiflexible polymers are particularly ubiquitous, being underlying mechanical components of biological systems such as cells or ECM, as well as many synthetic or biomimetic materials. Cell-derived components such as filamentous biopolymers or protein crosslinkers are readily available and well-studied model systems. However, as evolutionarily derived materials, they are constrained to a fixed set of structural parameters such as the rigidity and size of the filaments, or the valency and strength of binding of crosslinkers forming inter-filament connections. By implementing a synthetic model system based on the self-assembly of DNA oligonucleotides into nanometer-scale tubes and simple crosslinking constructs, we used the thermodynamic programmability of DNA hybridization to explore the impact of binding affinity on bulk mechanical response. Stepwise tuning the crosslinking affinity over a range from transient to thermodynamically stable shows an according change in viscoelastic behaviour from loosely entangled to elastic, consistent with models accounting for generalized inter-filament interactions. While characteristic signatures of concentration-dependent changes in network morphology found in some other natural and synthetic filament-crosslinker systems were not apparent, the presence of a distinct elasticity increase within a narrow range of conditions points towards potential subtle alterations of crosslink-filament architecture. Here, we demonstrate a new synthetic approach for gaining a deeper understanding of both biological as well as engineered hydrogel systems.

## Abstract 2

We introduce a new DNA-based approach to systematically investigate the bulk properties of crosslinked semiflexible DNA-based polymer networks as a model for natural biopolymer networks, by stepwise altering the binding affinity of the crosslinkers.

## Graphical abstract

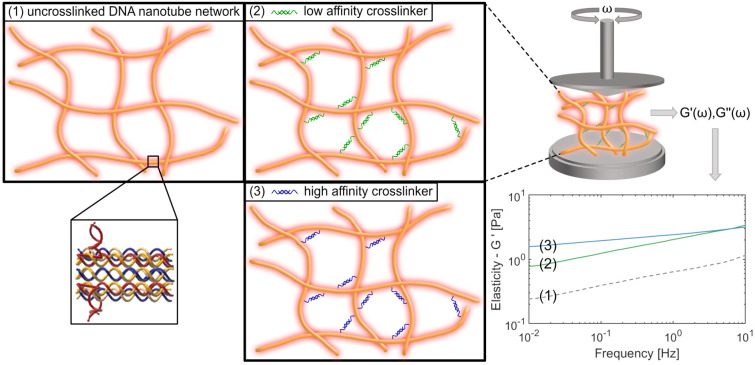

## 1. Introduction

Polymers are fundamental and highly versatile molecular building blocks of both synthetic and biological materials, ranging from commonly used plastics to living cells and complex biological matter. In contrast to synthetic polymers, which are typically based upon relatively simple structures of small, repeating units, such as the hydrocarbon chains in most plastic, biological polymers can be far more complex assemblies of protein-based building blocks. Well-studied examples are actin filaments, microtubules and intermediate filaments, which make up the underlying cytoskeleton of cells.^1^ This complex network is fundamental for the structural, mechanical, architectural and dynamic properties of cells.^1–6^ Based on their structural complexity, where individual monomers are typically globular proteins of several tens of kilodaltons in size, biopolymer networks can fulfil a very broad range of requirements necessary for living organisms. However, the nanoscale structure and properties of these biopolymers and in particular the molecular details of their accessory proteins such as crosslinkers can have profound impacts on the resulting macroscale properties. Therefore, for systematic investigations into their fundamental behaviours as materials, it is beneficial to decouple the additional influences of the different components in natural occurring networks by choosing a synthetic and minimalistic approach to stepwise model these type of systems.

From a mechanical perspective, most biopolymers such as actin and intermediate filaments are classified as “semiflexible”, expressing a persistence length *L*_p_, which is in the order of their typical contour length. Polymers assigned to this classification expresses thermally induced fluctuation modes while, unlike most synthetic flexible polymers, remaining an outstretched instead of a coiled configuration. These unique properties are crucial to provide stability but simultaneously enable dynamic processes^7^ as constituents of both extracellular structures^8^ and the intracellular cytoskeleton.^1–4^

Semiflexible biopolymers assemble into a wide range of different network architectures, depending on their conditions and associated crosslinking proteins.^9–11^ In the absence of further proteins, they assemble into entangled networks.^12–15^ In cellular systems, they usually occur in the presence of crosslinkers. Especially for actin, there is a wide range of well-studied crosslinkers,^9,10,16^ like the transient crosslinker α-actinin^17–19^ or the more permanent crosslinker fascin,^20,21^ which leads to the formation of higher ordered bundled structures. In some cases, small alterations to the binding properties of crosslinkers such as α-actinin have been implicated in the development of genetic disorders such as kidney disease.^22^ In relation to the engineering of biomaterials, chemical crosslinking, for example *via* electron beam radiation, can also be used to permanently alter and crosslink collagen networks.^23–25^ Reconstituted networks of filamentous actin (F-actin) are experimentally well-characterized and were used as an ideal for the development of theoretical models.^9,12,16,21,26,27^ The concentration-dependent scaling of the elastic plateau modulus, *G*_0_ ∝ *c*^7/5^, for entangled F-actin solutions is theoretically described within the frame of the tube model^28^ and has been experimentally verified.^12,29^ However, the theoretically predicted scaling with the persistence length, *G*_0_ ∝ *l*_p_^−1/5^, was contradicted in a DNA-based biopolymer system.^13^ Crosslinking is a mechanism that can be controlled by the cell to adapt to its environment and its mechanical properties. However, the exact details of how nanoscale properties such as binding strength, crosslinker size, crosslinker flexibility, and more impact the broader mechanical and morphological properties of bulk networks are still not fully understood. Theoretical models, like the affine model, are for example able to account for the nature of tightly crosslinked F-actin networks and predict the concentration dependent scaling of the elastic plateau modulus, *G*_0_ ∝ *c*^11/5^ correctly.^9,16^ However, capturing the rich parameter space that is given due to a variety of actin-associated proteins, such as actin binding proteins (ABPs), that form transient physical crosslinks between filaments, is beyond current capabilities.

With a considerable range in sizes, flexibilities, on–off rates and orientations, these crosslinks cannot be captured in a model that assumes permanent chemical connections, but rather build a bridge to entangled systems with pronounced, albeit non-permanent interactions between filaments. A central property that strongly influences the transient impact of crosslinks on the rheological properties of a material, but is still not represented in established models, is their binding affinity. Often, the investigation of isolated effects within biopolymer systems is impeded by the fact that naturally occurring proteins have many specific structural properties that cannot be altered in a decoupled fashion. Controlling the distance between the two binding domains of a transient crosslinker has been achieved through laborious protein engineering (for the case of filamin),^30^ however systematically fine-tuning the flexibility, and more importantly the affinity of binding domains to their target filaments is still a significant challenge.

**Fig. 1:**
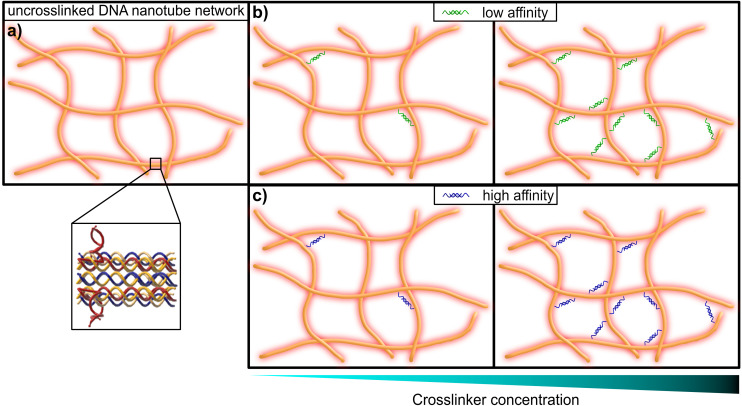
Graphical representation of a DNA nanotube network. (a) An entangled (uncrosslinked) network of DNA nanotubes. The inset shows the substructure of the double-crossover DNA nanotubes, that consist of partially-complementary single DNA strands, according to the original design by Rothemund *et al*.^31^ (b) DNA nanotube networks crosslinked by a DNA construct, that resembles a crosslinker with comparably low affinity (left: low concentration of crosslinkers, right: high concentration of crosslinkers). (c) DNA nanotube networks crosslinked by a DNA construct, that resembles a crosslinker with comparably high affinity (left: low concentration of crosslinkers, right: high concentration of crosslinkers).

**Fig. 2:**
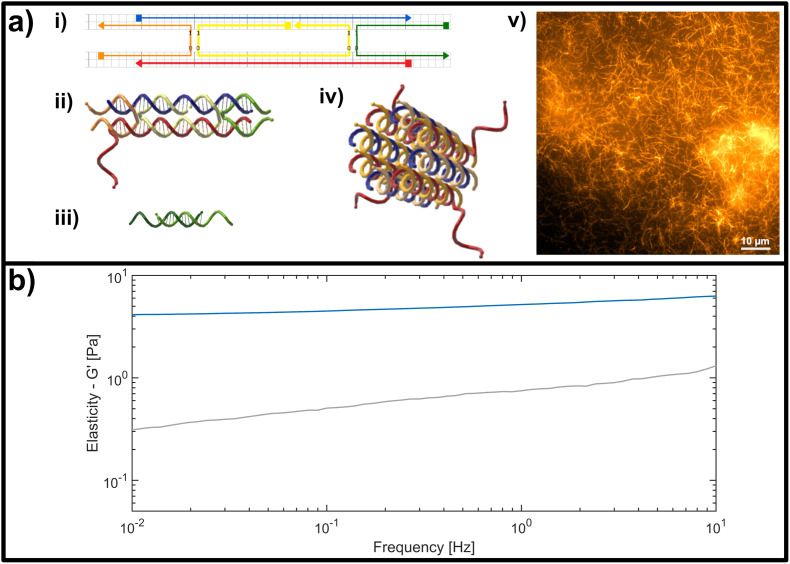
(a) Representation of the double crossover (DX) DNA nanotubes. (i) Schematic representation of the unmodified unit element consisting of five partially-complementary DNA strands, as first reported by Rothemund *et al.*^31^ (ii) Structural representation showing the interwoven strands, including an adenine (poly-A) single stranded overhang at the 3′-end of the red DNA sequence, used as a binding site for crosslinking. (iii) Crosslinking unit consisting of a 15 base-pair double-stranded middle segment with oligomeric thymine (poly-T) single-stranded DNA (ssDNA) overhangs with varying length on each side. (iv) Representation of a DNA nanotube formed by four unit elements. (v) Fluorescent image of a DNA-based nanotube network. (b) Representative measurement of the frequency-dependent response of an entangled (uncrosslinked) DNA nanotube network (grey line) and a fully crosslinked DNA nanotube network (blue line). Plotted is the dominant storage modulus *G*′, corresponding to the elasticity of the network.

This limitation can be circumvented exploiting the sequence-specific, thermodynamic binding properties of deoxyribonucleic acid (DNA). Schuldt *et al.* deployed a model system based on structural DNA programming which enabled a specific tuning of the filaments’ bending stiffness to isolate the impact of the persistence length on network rheology.^13^ In the present study, we leveraged the thermodynamic programmability of DNA-based materials, in order to systematically probe the impact of binding affinity for crosslinking elements in semiflexible networks of DNA nanotubes, in a range from so-called “transient” or low-affinity binding, to more stable high-affinity inter-filament links (see Fig. 1). Here we adapted the “double-crossover” DNA nanotube architecture originally developed by Rothemund *et al.*,^31^ where partially complementary single DNA strands were used to form three-dimensional hollow tubes (see Fig. 2a). The resulting DNA nanotubes have a mean length of 11.1 μm and a persistence length of 8.1 μm – this classifies them as semiflexible polymers (see ESI Fig. A2†).

The formation is initialized by a temperature gradient with a two-step formation: at higher temperatures between roughly 80 °C and 50 °C, the unit element is built (see Fig. 2a i,ii), which can be considered to be analogous to a monomeric unit. At lower temperatures of around 35 °C the subunits form elongated hollow, so-called “DX5 nanotubes” (see Fig. 2a iv).^32^ An earlier study conducted by Hariadi *et al.*^33^ shows that this two-step formation resembles similar assembly kinetics to biopolymers such as actin filaments or microtubules. This choice of nanotube architecture aims to mimic more closely the influence that crosslinkers have during the formation of a polymer network with comparison to natural occurring systems like actin.

A major advantage of using this system for studies on semiflexible polymer networks is the ease of their formation (see Fig. 2a v) and also their straightforward, modular modification.^34,35^ The functionality can be efficiently extended by adding modified versions of the single strands used for the original protocol to carry dye molecules or bio-active ligands.^36^ To experimentally address the impact of binding affinity on network mechanics, we modified the DX5 nanotubes with single stranded DNA overhangs to enable the formation of crosslinks through hybridization with bivalent constructs displaying pairs of complementary segments (see Fig. 2a iii), whose binding affinities depend on the overlap length of the respective single stranded DNA segments.

We systematically investigated the transition from entangled to crosslinked networks *via* bulk shear rheology on DX5 networks crosslinked with hybridized poly-A and poly-T overhangs of varying length, covering the range from low- to high-affinity binding. There are two well-studied crosslinking proteins associated with F-actin: α-actinin and fascin. Based on their experimentally determined dissociation constants of 0.4 μM^19^ to roughly 4.7 μM^37^ for α-actinin and approximately 150 nM for fascin,^38^ α-actinin can be considered as a low-affinity crosslinker whereas fascin represents a high-affinity crosslinker. The DNA-based crosslinkers have a poly-T overhang attached on the double stranded middle segment resembling the binding side with a length ranging from four to ten bases. Their associated dissociation constants can be calculated by estimation of their respective free Gibbs energy and the relation Δ*G* = *RT*ln *K* (see ESI Fig. A3†).^39,40^

This allows to tune the dissociation constants from values much smaller than one nM up to several μM, thus covering the range of the naturally occurring counterparts for actin filaments. Of course this comparison is further influenced by molecular details of the crosslinkers in comparison, as the length of the connecting segment between binding domains have been found to impact bulk mechanics,^30^ and more subtle factors such as flexibility of the connecting domain or relative angle between filament pairs at either end could also play some role. However, the synthetic crosslinkers described here can cover a wide range of *K*_D_-values and allow for a decoupled investigation of the binding affinity specifically.

We compared the complex shear modulus to the predictions of the glassy wormlike chain (GWLC) model. The GWLC, proposed by Kroy and Glaser,^41^ incorporates attractive interactions between filaments in an entangled network through the introduction of the stretching parameter *ε*, which has been shown to correlate to a generalized attraction or stickiness between filaments.^14,34^ Extending the ordinary wormlike chain (WLC), the minimal model to describe individual filaments, the assumption of a glassy surrounding in the GWLC is mathematically implemented as a stretching of the WLCs long-wavelength Eigenmodes with an Arrhenius-like exponential factor. The GWLC model has been successfully applied to explain non-specific interactions between filaments in different polymer model systems.^14,15,34,35^ Here we use the model to capture the slowdown of Eigenmode relaxation dynamics due to crosslinking of varying binding affinity.

## 2. Experimental

### 2.1. DNA nanotube formation

All relevant oligomers for hybridization of the DNA nanotubes were adapted from the sequences reported by Rothemund *et al.*^31^ and purchased *via* biomers.net with HPLC purification (see ESI Table A1†).^42^ To assemble a nanotube network of a specifically desired concentration, the required strands (SE1–SE5) were mixed in equimolar concentration in an assembly buffer containing 40 mM tris-acetate, 1 mM EDTA and 12.5 mM Mg^2+^ at a pH of 8.3. The concentration of each stock solution was confirmed *via* a Spectrophotometer NanoDrop 1000 (Thermo Fisher Scientifc Inc., USA) at a wavelength of 260 nm.

The nanotube networks were assembled by a thermal ramp adapted from the protocol from Ekani-Nkodo *et al.*^32^ In short, they were subjected to a temperature ramp in a TProfessional Standard PCR Thermocycler (Core Life Sciences Inc., USA), with the first step being denaturation for 10 min at 90 °C, and then complementary base pairing and assembly of the nanotube networks was achieved by lowering the temperature from 80 °C to 20 °C, at steps of 0.5 K every 10 min. After hybridization, DNA nanotubes were stored at room temperature.

For visualization the oligomer SE3 was modified with the fluorescent Cyanine dye 3 (SE3-Cy3) with two additional spacer thymine bases (T–T) in between the main part of the sequence incorporated into the tube and the dye itself. DNA nanotubes were labelled by partially or fully replacing the unlabelled oligo SE3 by SE3-Cy3. The crosslinkers designed for this study are based on a 15 base-pair complementary section in the centre and poly-T overhangs at each end ranging from 4 to 10 thymines (see Fig. 2a). After denaturation at 90 °C for 10 min, crosslinkers were hybridized by a stepwise isothermal holding at 53 °C, 48 °C and 43 °C for 10 min each and then stored at 4 °C. Their comparable yield and purity was confirmed by a PAGE Gel (see ESI Fig. A1†). For rheological measurements, the hybridized DNA nanotubes were placed on the rheometer and allowed to equilibrate for 1 h, before frequency and strain dependent measurements were performed.

### 2.2. Imaging techniques

Fluorescence imaging was carried out using an *epi*-fluorescence Leica DM IRB microscope equipped with a 100× oil-immersion objective (Leica 11506168) and an iXon DV887 back illuminated EMCCD camera (Andor Technology). Fluorescence excitation was induced with a mercury vapor lamp and a N2.1 filter cube (Leica 11513882, excitation filter from 515 to 560 nm) transmitting only the wavelength exciting Cy3 to the sample. Images were recorded as grayscale pictures with the camera-associated Andor SOLIS software. For determination of the persistence length and contour length filaments were absorbed to a glass surface. For the persistence length evaluation it was ensured that the absorbed filaments were not influenced by surface-filament interactions *via* kurtosis analysis as previously described.^13^

### 2.3. Shear rheology

Shear rheology measurements were performed with a strain controlled ARES rheometer (TA Instruments, USA) equipped with a 25 mm cone-plate geometry at a gap width of 50 μm. 175 μl of pre-hybridized DX nanotubes were carefully placed on the rheometer and allowed to equilibrate for 1 h at 20 °C or 25 °C, respectively. To minimize evaporation during measurement samples have been surrounded with a custom-made humidifier, containing a water reservoir and wet sponges. During equilibration a dynamic time sweep with measurements every 2 min at frequency of 1 Hz and a strain of 5% was performed. Data was recorded with a dynamic frequency sweep ranging from 0.01 Hz to 10 Hz at a strain of 5%. Further data analysis was performed with a self-written MATLAB script (MathWorks, USA) (Wolfram Research, USA) and a self-written Python script.

## 3. Results and discussion

### 3.1. Influence of crosslinker affinity on DNA nanotube networks

The DX5 nanotube system and its components have mechanical properties that resemble the semiflexible biopolymer F-actin, however they can be modified in a straightforward manner according to simple rules of DNA-based nanofabrication. Equipping DX5 nanotubes with single-stranded DNA (ssDNA) overhangs enables a simple strategy for hybridization-based crosslinking, where the binding affinity of the filament-binding domains (complementary ssDNA sequences) depending on the sequence and overlap length of complementary base-pairing. Here, for the sake of simplicity of design and synthesis, we used oligomeric adenine (poly-A) and thymine (poly-T) sequences as the complementary, overlapping domains on the DX5 nanotube filaments and crosslinkers, respectively (see Fig. 2a). For crosslinking, the SE5 strand of the nanotubes was appended on the 3′ end with a 15-base poly-A overhang to act as the universal binding point. The crosslinking constructs were formed by a 15-base dsDNA segment, flanked on both ends with variable-length, ssDNA, poly-T overhangs extending from the 3′ end of each strand. This allowed the binding strength of the domains, and ultimately the strength of crosslinking interaction between filament pairs, to be solely governed by the length of the hybridized poly-A/poly-T segment, enabling us to systematically investigate which impact the binding affinity on the molecular level has on the bulk, mechanical signatures of semiflexible polymer systems.

In order to capture the influence of crosslinker binding affinity on the viscoelastic properties of networks, bulk shear rheology experiments were performed at room temperature on unmodified, purely entangled DX5 nanotube networks as well as on versions modified to include crosslinking interactions of varying strength. To roughly compare the relative binding strength of each crosslinker, we calculated the Gibbs free energy using the OligoAnalyzer tool from IDT.^39^ The shortest, and thereby most weakly-binding crosslink segment used in this study consists of four thymine bases, which corresponds to a binding strength of roughly 6 kcal mol^−1^, whereas the longest was ten bases, corresponding to around 18 kcal mol^−1^. The Gibbs free energy scales linearly with the number of A–T base-pairs formed between the central crosslinking construct and the binding point on the DX5 nanotube filament (see ESI Fig. A3†). In comparison to commonly studied biological crosslinkers for actin filaments, these values can be transferred into dissociation constants ranging from values well below one nM up to several μM (see ESI Fig. A3†).

**Fig. 3:**
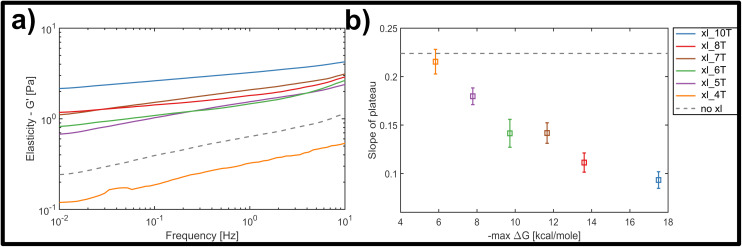
(a) Average frequency-dependent storage modulus of crosslinked DNA nanotube networks with differing crosslinker affinity, measured by bulk shear rheology. For increasing crosslinker affinity at a constant crosslinker concentration of 2 μM, the measured plateau flattens and increases in its magnitude. The average response of an entangled network is given by the gray dashed line. (b) With increasing length of the crosslinker overhang and thus increasing binding affinity, estimated by the maximal free enthalpy, the average slope of the plateau in the frequency-dependent elastic modulus decreases.

Fig. 3a displays the bulk storage modulus *G*′ (*e.g.* elasticity) for the uncrosslinked reference system (grey, dashed line) and the networks crosslinked with links of successively increasing binding affinity. Unsurprisingly, we found a general increase in elasticity upon increase of crosslink binding affinities, when a single, molar ratio of 1 to 10 crosslinking constructs per binding point on the filaments was tested. The shortest version of the crosslinks did not result in network stiffening but rather weakened the elastic properties. While at first counterintuitive, a mild weakening effect arising from small amounts of crosslinking has been observed in actin networks crosslinked by either natural proteins or synthetic DNA-peptide chimeras,^9,10,43^ and has been attributed to local heterogeneities triggering a global softening at the onset of filament–filament interactions.

Nevertheless, the overall trend, as well as a minimum threshold of 5 matching base-pairs for any global stiffening effect found in this study suggests that there is a minimum binding affinity that has to be exceeded in order for any increases in filament–filament interactions to percolate throughout the network (see Fig. 3a). For all crosslinker strengths, we observed a plateau-like region in the frequency-dependent elastic modulus that exhibited a weak power-law behaviour with exponents *α* between *α* = 0 and *α* = 0.5. Fig. 3b shows the decline of average slope values from 0.22 for uncrosslinked DX5 nanotubes, to 0.08 for networks crosslinked by 10 A–T base-pairs. This corresponds to an increase of the inter-filament interactions, *i.e.* higher stickiness, which is induced by the stronger crosslinking. This also indicates a smooth transition from more fluid-like (uncrosslinked) to more rubber-like (crosslinked) networks for increasing binding affinities. As a comparison, natural occurring semiflexible biopolymer networks consisting of purely entangled actin have previously been reported to express a power-law exponent of 0.14. Vimentin networks, which are known to exhibit far stronger and more “sticky” filament-filament interactions show an exponent of 0.07.^34^

### 3.2. Concentration-dependent behaviour of transient and stable crosslinkers

We examined the influence of crosslinker concentration on the mechanical signatures of the DX5 filament networks for two distinct binding affinities; one representing the weakly-binding, transient regime (6 hybridized A–T base-pairs) and the other the more stable interaction (10 A–T base-pairs), when measured at room temperature (20 °C). Here, the aim was to assess typical signatures of affinely crosslinked networks in the strong-binding case,^9,16^ namely a progressive flattening of the plateau and shift to a more stretch-driven, rubber-like network. For each crosslinker type we tested its stiffening impact on network rheology in molar ratios of crosslinks to potential binding points on the filaments ranging from 1 : 500 to 1 : 5, which corresponds to concentrations from 0.04 μM to 4.00 μM. To ensure an even distribution of crosslinks over the sample and allow for equilibration, homogenization was induced by increasing the temperature by 5 K above room temperature to 25 °C, still well below the temperature at which DX5 nanotube structures are significantly impacted (see ESI Fig. A5†). After this equilibration, measurements were performed at 20 °C.

**Fig. 4:**
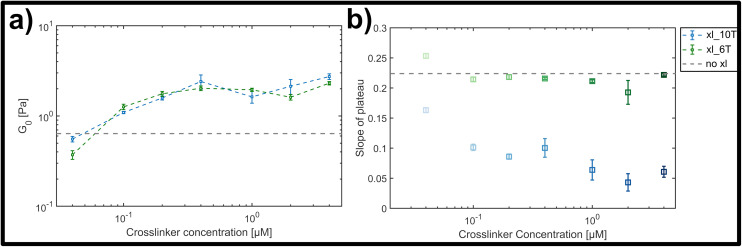
(a) Average storage modulus *G*_0_ for lower (green) and higher (blue) crosslinker affinities, formed by a hybridized A–T segment of either 6 or 10 base-pairs in length. (b) Slope of plateau derived from the frequency-dependent storage modulus for the lower (green) and the higher (blue) crosslinker affinity (see ESI Fig. A4† for frequency dependent curves). While for the lower affinity the slope stays almost constant, there is a clear drop of the slope with increasing concentration for the higher affinity.

The magnitude of the elastic plateau modulus over a frequency range from 0.01 to 10 Hz showed a similar, gradual increase upon crosslinker concentration increase for both transient and stable crosslinker types (see ESI Fig. A4†). This general trend is clear when comparing the average elastic plateau modulus *G*_0_, given by the elasticity at a frequency of 1 Hz, for the two crosslinker types over the measured concentration range (see Fig. 4a). In both cases a potentially triphasic signature of *G*_0_ as a function of crosslinker concentration was observed. Here, this was seen through an initially linear, monotonic increase of *G*_0_ in the low concentration range (0.04 μM–0.4 μM), followed by a plateau or possible slight drop at moderate concentrations (0.4 μM–2 μM), and finally an increase at the highest concentration measured (4 μM). This behaviour is similar to prior observations in crosslinked F-actin networks, albeit far less pronounced here.^9,10^ In prior studies, this was attributed to concentration-dependent structural polymorphism, *i.e.*, changes in the network morphology due to gradual bundling of filaments into fibres – however is possibly diminished in this case due to molecular or geometric details of the crosslinkers and the lattice of binding points on the DX5 filaments.

While both the relative magnitude and increase of *G*_0_ as a function of crosslinker concentration were similar for both transient and stable types, the main difference observed was in the resulting power-law behaviour of frequency-dependent slopes. This can be qualitatively seen in the individual frequency sweeps depicted (see ESI Fig. A4†), where Fig. A4(i)† shows the elastic plateau of the more transient crosslinker (6 A–T base-pairs) and Fig. A4(ii)† shows the plateau for the more stable crosslinker (10 A–T base-pairs). In each panel, the transition from a lighter to a darker colour indicates the increase in crosslinker concentration, and the general concentration-dependent stiffening effect already depicted in Fig. 4a can be seen. However, for the 10 A–T base-pair crosslinker, a successive flattening of the slope with increasing concentration is evident compared to the transient crosslinks. This evolution of the slope is plotted in Fig. 4b, where a clear decrease is seen for even moderate concentrations of the stable crosslinker before reaching stable minimum of 0.05, in contrast to a constant slope of approximately 0.22 for the transient variant. This behaviour indicates a clear transition from a bending-dominated response to an affine stretching of the entire network^16^ when stable, albeit noncovalent interactions are imposed between individual filaments.

In order to quantify this slowdown of Eigenmode relaxation dynamics due to crosslinking, we evaluated the rheology data within the GWLC model in order to quantify “sticky” filament-filament interactions through the stretching parameter *ε*. The model accounts for sticky interactions by a stretching of the filaments’ mode relaxation times *τ*_*λ*_ > *τ*_*Λ*_ of all Eigenmodes of (half) wavelength *λ* longer than a characteristic interaction length *Λ*. These are stretched with the factor *e*^*εN*^, where *N* = *λ*/*Λ* − 1 describes the interactions per wavelength *λ*. The stretching parameter *ε* can also be interpreted as a measure for the decrease of mode relaxation dynamics that results from transient crosslinking. A more detailed description of this model is presented in the ESI† (section ‘Glassy wormlike chain analysis’).

We analysed the rheology data by fitting the storage modulus *G*′(*f*) to the mean curves of the measured data with
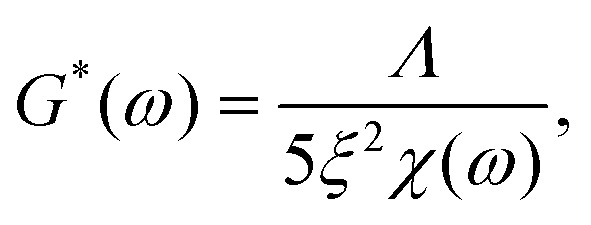
where *ω* = 2π*f* and *χ*(*ω*) is the micro-rheological response function of the GWLC to a point force at its ends. The fitting routine was implemented in a self-written Python script (and overview of the fitting parameters is shown in ESI Table A2†). As we investigated DX5 nanotube networks of same concentrations, we chose the same parameters for the description of individual filaments as well as the same network defining parameters. Fitting the model to the experimental data we consistently obtained *ε* values around *ε* = 6 for all but the lowest concentration (where we found *ε* = 1.3) for the transient, 6 A–T base-pair crosslinker. This corresponds to roughly double the value for the uncrosslinked reference network where the model yielded *ε* = 2.5. For the stable, 10 A–T base-pair crosslinker, the *ε* value increases gradually upon concentration increase from *ε* = 2.3 to *ε* = 17.9.

In contrast to the transient crosslinker, this constitutes a remarkable slowdown of relaxation dynamics that results from strong attractive interactions or permanent connections between filaments. A similar behaviour was previously shown for composite keratin/actin networks, where the *ε* value underwent a drastic increase with decreasing actin content.^15^

### 3.3. Temperature-dependent variation of plateau modulus

We finally investigated the impact of varying the temperature at which the system was initially homogenized prior to rheology measurements, in order to determine how potential heat-induced rearrangements of crosslinkers within the network changed the mechanical response for the different overlap lengths. This was practically limited by the known approximate melting temperature of the nanotubes at approximately 35 °C.^32^ This is the temperature where thermal energy overcomes the hybridization of the five-base overlaps and pi-stacking interactions holding the individual 5-strand subunits depicted in Fig. 2a bound to each other, and can be viewed as the threshold for a temperature-dependent polymerisation and depolymerisation transition. Due to the relatively low, and broad range of melting temperatures of even long segments of A–T hybridization, we do expect a general homogenization of crosslinks within the network at temperatures below the 35 °C threshold for filament depolymerisation. However, we cannot exclude that there might be some differences in terms of the percentage of crosslinkers that are bridging two distinct filaments compared to those that are bound to two neighbouring sites on the same filament.

**Fig. 5:**
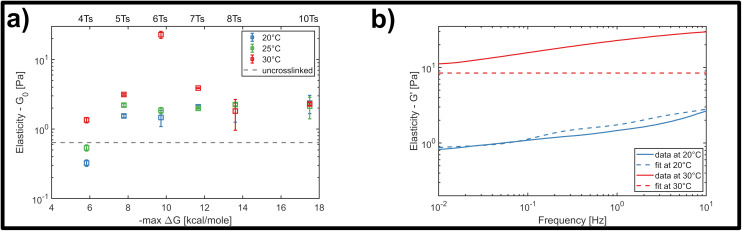
(a) Temperature-dependent response of the crosslinked DNA nanotubes to bulk shear rheology at a fixed frequency of 1 Hz, and at a fixed crosslinker concentration of 2 μM. For higher homogenization temperatures, additional effects influence the elasticity of the material drastically, increasing *G*_0_ by a factor of 10. (b) Representative fit of the storage modulus of the GWLC model with and without a temperature change. The model can predict the behaviour at room temperature, but fails to predict the exemplary curve for a homogenization temperature of 30 °C.

Therefore, we examined the mechanical response for each of the six crosslinker variants (4, 5, 6, 7, 8 and 10 A–T base-pairs), at a constant crosslinker concentration of 2 μM, comparing homogenization temperatures of 20 °C, 25 °C and 30 °C. Fig. 5a shows the stepwise network stiffening for all investigated crosslinker types. For shorter crosslinkers (4, 5, 6 and 7 base-pairs), a general stiffening of the network resulting from a higher homogenization temperature was observed through the increase in *G*_0_, however this was absent for the two longest crosslinkers (8 and 10 base-pairs). In most cases, the impact of the stiffening was moderate, accounting for approximately a 2-3-fold increase in *G*_0_, however a significant ten-fold jump in *G*_0_ was consistently found specifically for crosslinkers consisting of six A–T base-pairs. This sudden temperature-dependent jump at an intermediate crosslinker strength of 6 base-pairs indicates that subtle changes in the configurational distribution of crosslinkers in the network has a strong influence on the global mechanical properties. In this case, it is very likely that heat induced scission events and stochastic rebinding have profound consequences for the network connectivity.

In general, the moderate stiffening effect observed as a result of variations in the homogenization temperature can be accounted for within the framework of the GWLC model, which suggests enhanced filament-filament attractions within the network. This is possibly caused a subtle shift towards a greater population of crosslinkers bridging pairs of distinct, neighbouring filaments compared to those binding multiple sites the same filament. However, the drastic ten-fold increase in elasticity that is observed for the rather transient 6 base-pair crosslinker cannot be explained by the theory, regardless of the choice of parameters (see Fig. 5b and ESI Fig. A6 and A7†). This is possibly due to the fact that the GWLC model is designed to explain mutual interactions between filaments in a homogeneous, entangled network of semiflexible polymers. The limitations of the GWLC have been demonstrated in previous studies on networks of the cytoskeletal intermediate filament keratin^15,24,34^ and were explained with network architectures which were incompatible with the prerequisites of the theory. We suspect that the inability to account for the jump for the 6 base-pair crosslinkers in this case has a similar cause.

## 4. Conclusion

The state space of crosslinks in networks of semiflexible polymers typically cannot be simply reduced to “bound” and “unbound” in a binary way. In fact, through some changeable parameters, natural crosslinkers offer a variety of binding affinities, which greatly enriches the range of possible mechanical behaviours in a bulk network beyond a simply altering the magnitude of *G*_0_. The DNA-based approach presented here is ideal for the systematic investigation of crosslinker parameters such as binding affinity, since its influence on network rheology can be specifically investigated in an isolated fashion by simply making alterations to the length and sequence of hybridized segments forming the noncovalent link between individual DNA-based semiflexible filaments. This is an elegant way to test the validity of proposed physical laws and models such as the GWLC, and to propose extensions if necessary. It also further emphasises the broad functionality that DNA-based hydrogels have to offer.^44–46^

Our experiments showed a clear influence of binding affinity on several aspects of the resulting network properties. The slope of the frequency dependence of the elastic modulus clearly decreased for increasing binding affinity, suggesting a transition to rubber-like elasticity consistent with stronger filament-filament interactions. This gradual transition cannot be explained by any established theory for entangled or crosslinked networks of semiflexible filaments, whether it be the affine model established for crosslinked actin filaments,^16^ or the tube model for entangled networks.^12^ To some extent, this changing power-law behaviour can be explained within the framework of the GWLC, by factoring in the crosslinkers, particularly the stronger, less transient varieties, as a progressive increase in “sticky” filament-filament interactions. Nevertheless, potential changes in underlying network morphology induced by temperature-triggered re-distribution of crosslinker configuration in the 6 base-pair variant caused an increase in *G*_0_ that deviated strongly from the structural assumption of the GWLC model, which could not be analytically analysed. Consequently, a more comprehensive theoretical model is likely needed to account for non-homogeneous network morphologies. This model must address the nature of the filament-filament coupling by application of crosslinks to explain the emergence of elastic bulk properties.^27^

Using DNA-based nano-fabrication to mimic naturally occurring biopolymers opens a huge range of future opportunities for extending the work presented here. In our proof-of-concept study, our strategy for varying the binding affinity was solely based upon the simple case of altering the length of the poly-T overhang of the crosslinkers, which then hybridised *via* complementary base-pairing to poly-A overhangs on the DX5 nanotube filaments. However, it would also be feasible to alter the G–C content within this hybridised region between the overhang and filament, while keeping its length constant to alter the binding affinity. This would enable an extraordinary ability to precisely fine-tune the binding affinity for any given length of N paired bases. Since not only the absolute ratio of A–T *versus* G–C pairs impacts the binding energies between strands, but their sequence-specific arrangement also plays a role due to stacking interactions between neighbouring bases,^47,48^ there are potentially 4^N^ unique binding affinities theoretically available between the boundary cases of poly-A–T and poly-G–C base-pairing. Furthermore, following the earlier work of Wagner *et al.*,^30^ effects arising from variation of the inter-filament distance imposed by the crosslinker could be investigated by varying the length of the central paired region, as could the impact of linker flexibility through the incorporation of mis-paired joints.

Perhaps most interestingly from the standpoint of biological soft matter, earlier theoretical modelling of strongly crosslinked, affine, semiflexible networks suggests that the persistence length of the individual biopolymers would also be a key factor in bulk network behaviour, due to the bending of filament segments and stretching out of thermal fluctuations between stable crosslink points.^16^ This previously inaccessible interplay between filament and crosslinker properties could be explored by altering the underlying DNA nanotube design to allow for variations in their persistence length *L*_p_, similar to previous work.^13,49^ This opens another interesting parameter space, where in contrast to the decoupled investigation of the binding affinity of the crosslinkers, the mechanical alteration of the underlying network could be simultaneously addressed.

## Author contributions

Martin Glaser carried out experimental work, performed data analysis, conceptualised and wrote the manuscript. David M Smith and Jörg Schnauß conceptualised, wrote and edited the manuscript. Dusan Prascevic carried out experiments and edited the manuscript. Paul Mollenkopf performed data analysis and edited the manuscript. Catarina Ferraz prepared figures, wrote and edited the manuscript. Josef A. Käs conceptualised and edited the manuscript.

## Conflicts of interest

There are no conflicts to declare.

## Supplementary Material

NR-015-D2NR05615A-s001
