# Supplementary material for: Systematic altering of semiflexible DNA-based polymer networks via tunable crosslinking
Source: Nanoscale. 2023 Apr 5;15(16):7374–83. doi: 10.1039/d2nr05615a (PMC10134436; doi:10.1039/d2nr05615a)
Supplement: NR-015-D2NR05615A-s001 [file NR-015-D2NR05615A-s001.pdf]

Electronic Supplementary Material (ESI) for Nanoscale.
This journal is © The Royal Society of Chemistry 2023

Appendix

Name	Sequence (5’ to 3’ end)
SE1	CTCAGTGGACAGCCGTTCTGGAGCGTTGGACGAAACT
SE2	GTCTGGTAGAGCACCACTGAGAGGTA
SE3	CCAGAACGGCTGTGGCTAAACAGTAACCGAAGCACCAACGCT
SE3-Cy3	CCAGAACGGCTGTGGCTAAACAGTAACCGAAGCACCAACGCTTT-Cy3
SE4	CAGACAGTTTCGTGGTCATCGTACCT
SE5	CGATGACCTGCTTCGGTTACTGTTTAGCCTGCTCTAC
SE5_15A	CGATGACCTGCTTCGGTTACTGTTTAGCCTGCTCTACAAAAAAAAAAAAAAA
xLfw_15bp_4T	AGCAGGACCTCACCATTTT
xLrv_15bp_4T	TGGTGAGGTCCTGCTTTTT
xLfw_15bp_5T	AGCAGGACCTCACCATTTTT
xLrv_15bp_5T	TGGTGAGGTCCTGCTTTTTT
xLfw_15bp_6T	AGCAGGACCTCACCATTTTTT
xLrv_15bp_6T	TGGTGAGGTCCTGCTTTTTTT
xLfw_15bp_7T	AGCAGGACCTCACCATTTTTTT
xLrv_15bp_7T	TGGTGAGGTCCTGCTTTTTTTT
xLfw_15bp_8T	AGCAGGACCTCACCATTTTTTTT
xLrv_15bp_8T	TGGTGAGGTCCTGCTTTTTTTTT
xLfw_15bp_10T	AGCAGGACCTCACCATTTTTTTTTT
xLrv_15bp_10T	TGGTGAGGTCCTGCTTTTTTTTTTT

Table A1: List of used DNA oligonucleotides as purchased from biomers.net with HPLC purification.[1]

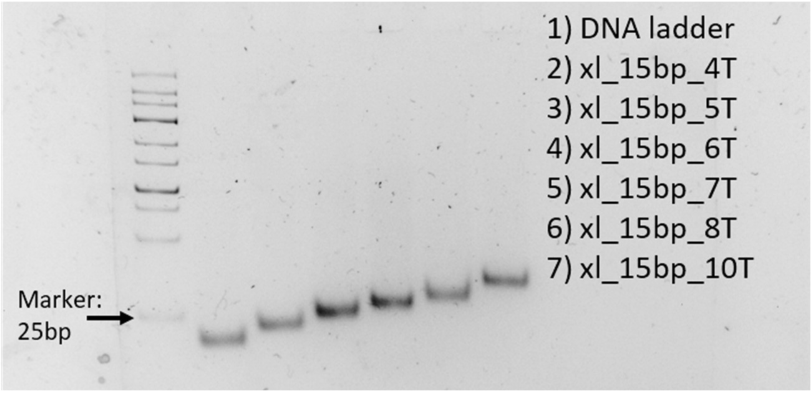

Figure A1: Yield and purity of the designed crosslinkers was confirmed via a native 12% (v/v) PAGE gel.

Polyacrylamide gel electrophoresis (PAGE) was performed using a Mini-PROTEAN® Tetra cell system (Bio-Rad, Germany) and its related components (Bio-Rad, Germany). Polyacrylamide was mixed with water and TBE buffer (final concentration 89 mM tris base, 89 mM boric acid, 2 mM EDTA) and 0.1% (v/v) N,N N´,N´- Tetramethylethylenediamine (TEMED,Carl Roth, Germany) and 0.1% (w/v) Ammonium persulfate (Carl Roth, Germany) were added. Gel polymerization took place between two glass plates for approx. 30 min with a comb inserted to form sample pockets. The formed gel is immersed in running buffer (0.5x TBE). As an indicator for the sample sizes a marker (GeneRuler 1 kb DNA Ladder, Thermo Fisher Scientific, USA) was used. Pre-hybridized crosslinkers containing 6x DNA Gel Loading Dye (Thermo Fisher Scientific, USA), were applied to the sample pockets. The electrophoresis was performed at 120 V for approx. 1.5 h at room temperature using PowerPac™ Basic Power Supply (Bio-Rad, Germany). A dilution of 1x SYBR™ Gold nucleic acid gel stain (Life Technologies, USA) in 0.5x TBE was used to stain the gel for approx. 20 min before imaging. SYBR™ Gold exhibits fluorescence enhancement upon binding to both ssDNA and dsDNA. Gels were imaged using the ChemiDoc MP Imaging System with UV excitation (302 nm) and 590/110 nm emission.

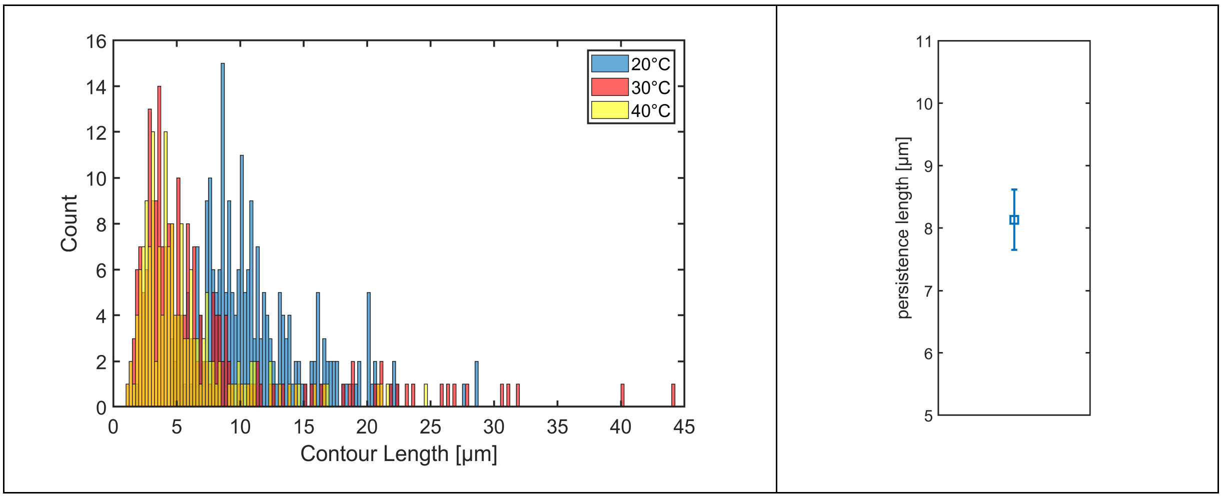

Figure A2: Left: Distribution of the measured contour length of individual filaments when equilibrated at 20°C, 30°C and 40°C for 1h. The respective mean contour length is: 11.1 µm at 20°C, 7.4 µm at 30°C and 5.8 µm at 40°C. Right: The evaluation of the persistence length at room temperature has given a mean value of 8.1 µm.

For determination of the persistence length and contour length filaments were adsorbed to a glass surface and were allowed to equilibrate for 1h at room temperature. For the persistence length evaluation a kurtosis analysis, as previously described,[2] was used to ensure that the adsorbed filaments taken into account were not influenced by surface-filament interactions.

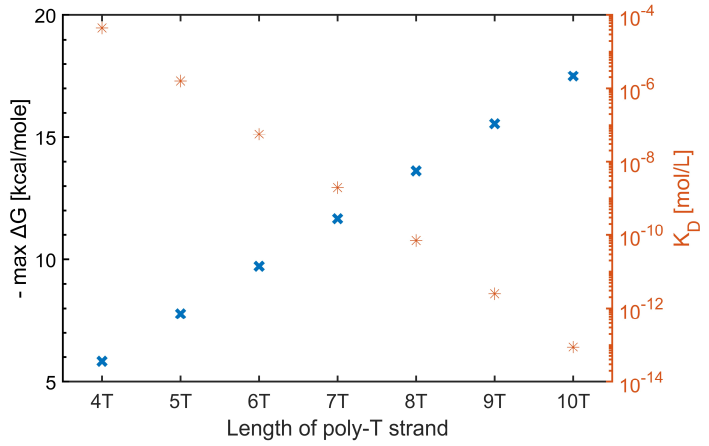

Figure A3: Relation of the length of the poly-T overhang on the crosslinker to: (left y-Axis) the estimated molar Gibbs free energy, evaluated using the OligoAnalyzer online tool from IDT[3] and (right y-Axis) the calculated dissociation constant with the relation $\Delta G=RT\ln K_D$.[4]

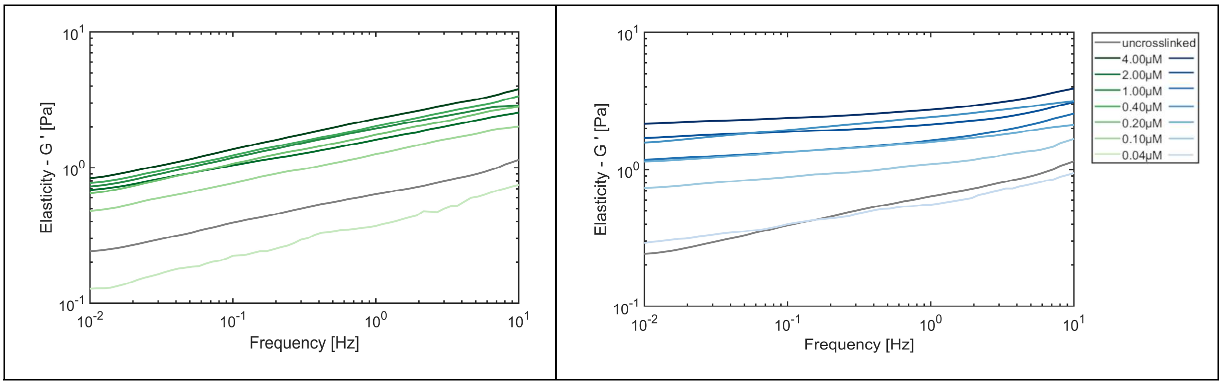

Figure A4: Average frequency-dependent storage modulus for (left) a lower and (right) a higher crosslinker affinity, formed by a hybridized A-T segment of either 6 or 10 base-pairs in length. In each panel the concentration of the applied crosslinkers increase with darkening of the respective color.

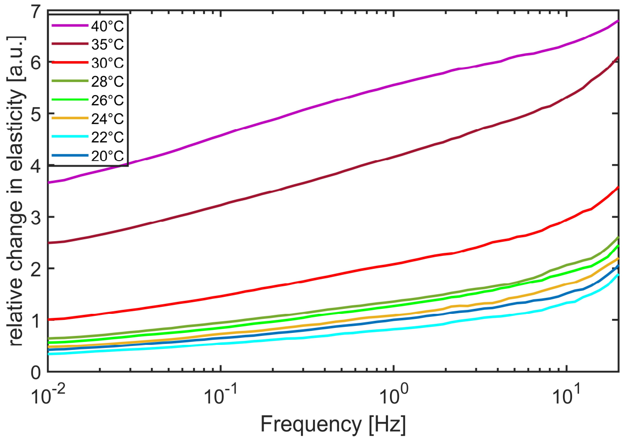

Figure A5: Frequency dependent relative change of the storage modulus of uncrosslinked DX5 nanotube networks for different equilibration temperatures. Above 30°C a drastic change of the network behaviour can be observed, indicating a rearrangement of the underlying network architecture itself.

Glassy wormlike chain (GWLC) analysis

Fit parameter for GWLC fit	value
persistence length	l_p = 8,1 µm (see fig. A2)
contour length @ 20°C	L_c = 11.1 µm (see fig. A2)
contour length @ 30°C	L_c = 7.4 µm (see fig. A2)
contour length @ 40°C	L_c = 5.8 µm (see fig. A2)
mesh size	ξ = 0.36 µm[5]
interaction length	Λ = 1.0 – 1.8 µm
drag coefficient per length	$\zeta_\perp$ = 2mPa s[5]

Table A2: Fit parameters used for the GWLC analysis.

The GWLC model used in this study has been shown to give a more robust theoretical description for the behavior of semiflexible polymer systems.[5–7] The GWLC is a theoretical model, that provides an extension to the wormlike chain (WLC) model for semiflexible polymer networks. The mode relaxation spectrum of the WLC is stretched exponentially to account for glassy interactions of a test chain within its polymer background. Time for mode relaxation of all Eigenmodes of (half-) wavelength λ_n = L/n and mode number n for a WLC (with persistence length l_p and the transverse drag coefficient $\zeta_\perp$) are given by:

$$\tau_n^{WLC} = \zeta_\perp / (\frac{l_p\, k_B\, T\, \pi^4}{\lambda_n^4} + f\, \pi^2 / \lambda_n^2).$$

Therefore, the relaxation times of the GWLC are modified according to:

$$\tau_n^{GWLC} = \begin{cases} \tau_n^{WLC} & if\ \lambda_n \leq \Lambda \\ \tau_n^{WLC} e^{\varepsilon N_n} & if\ \lambda_n > \Lambda, \end{cases}.$$

Here, $N_n = \lambda_n / \Lambda - 1$ is the number of interactions per length λ_n. Λ represents the average distance between interaction points. L is the contour length of the test filament. ε, the stretching parameter, gives the magnitude of how strong the modes are slowed down by interactions with their background. f describes a homogeneous backbone tension accounting for existing pre-stress. The complex linear shear modulus in the high frequency regime is given by:

$$G^*(\omega) = \Lambda/(5\xi^2 \chi(\omega)).$$

with ξ being the meshsize of the network. The micro-rheological, linear response function χ(ω) to a point force at the ends of the GWLC is calculated as:

$$\chi(\omega) = \frac{L^4}{\pi^4\, l_p^2\, k_B\, T} \sum_{n=1}^{\infty} \frac{1}{(n^4 + n^2 f / f_E)(1 + i\omega\tau_n^{GWLC}/2)}.$$

Here, f_E is the Euler buckling force $f_E = l_p k_B T \pi^2 / L^2$. For the linear regime presented in this study f is set to zero.

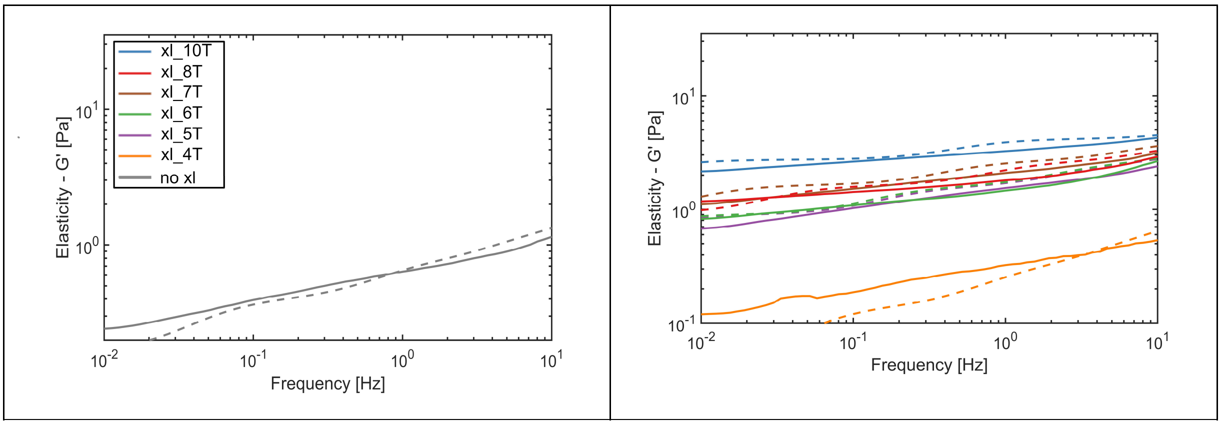

Figure A6: The GWLC fits (dashed line) for an uncrosslinked network (left) and for the different crosslinker affinities (right) give a valid approximation for the measured data sets (solid line).

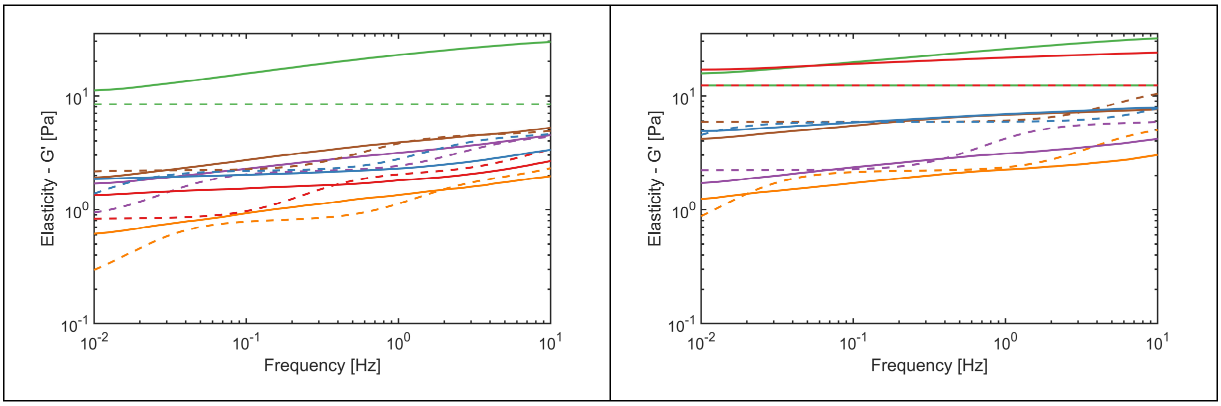

Figure A7: The GWLC fits (dashed line) for conditions at preheating of 30°C (left) or 40°C (right), show that the model predictions for the measured data sets (solid line) collapse, if the networks undergo internal structural, architectural or morphological changes.

References

1 Oligos, DNA / RNA-Synthese - biomers.net Oligonucleotide, https://biomers.net/, (accessed 5 October 2022).

2 C. Schuldt, J. Schnauß, T. Händler, M. Glaser, J. Lorenz, T. Golde, J. A. Käs and D. M. Smith, *Phys. Rev. Lett.*, 2016, **117**, 197801.

3 Oligo Analyzer, https://eu.idtdna.com/calc/analyzer, (accessed 4 October 2022).

4 J. Pääkkönen, J. Jänis and J. Rouvinen, *ACS Omega*, 2022, **7**, 10789–10795.

5 T. Golde, M. Glaser, C. Tutmarc, I. Elbalasy, C. Huster, G. Busteros, D. M. Smith, H. Herrmann, J. A. Käs and J. Schnauß, *Soft Matter*, 2019, **15**, 4865–4872.

6 T. Golde, C. Huster, M. Glaser, T. Händler, H. Herrmann, J. A. Käs and J. Schnauß, *Soft Matter*, 2018, **14**, 7970–7978.

7 I. Elbalasy, P. Mollenkopf, C. Tutmarc, H. Herrmann and J. Schnauß, *Soft Matter*, 2021, **17**, 3954–3962.
